# Supplementary material for: Dysregulation of the Enteric Nervous System in the Mid Colon of Complement Component 3 Knockout Mice with Constipation Phenotypes
Source: Int J Mol Sci. 2022 Jun 20;23(12):6862. doi: 10.3390/ijms23126862 (PMC9225043; doi:10.3390/ijms23126862)
Supplement: Supplementary file 1 [file ijms-23-06862-s001.zip › ijms-1697364-supplementary.pdf]

Supplement Figure

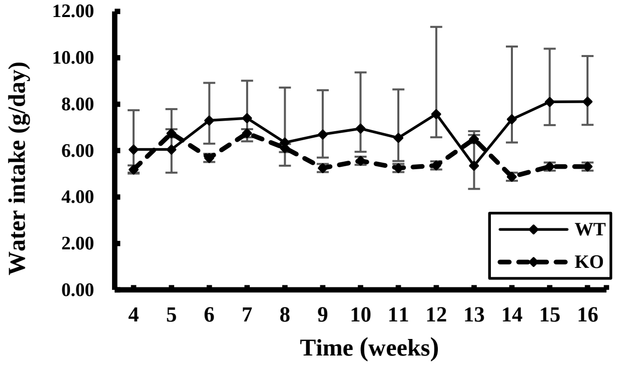

(a)

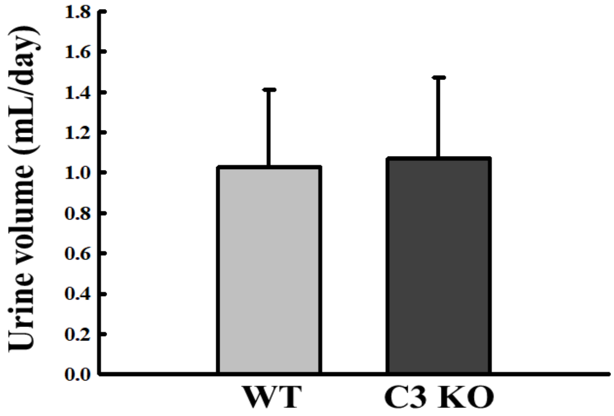

(b)

Supplement Figure S1. Water intake and urine volume. (a) Water intake of WT and C3 KO mice. The volume of water intake per day were measured using a measuring cylinder, and then average level was calculated based on these data. (b) Urine volume of WT and C3 KO mice. After collection of urine from metabolic cage, the volume of urine was measured using a measuring cylinder, and then average level was calculated based on these data.
